# Preparation of Hop Estrogen-Active Material for Production of Food Supplements

**DOI:** 10.3390/molecules26196065

**Published:** 2021-10-07

**Authors:** Marcel Karabín, Tereza Haimannová, Kristýna Fialová, Lukáš Jelínek, Pavel Dostálek

**Affiliations:** Department of Biotechnology, Faculty of Food and Biochemical Technology, University of Chemistry and Technology, Technická 5, 166 28 Prague 6, Czech Republic; marcel.karabin@vscht.cz (M.K.); tereza.haimannova@gmail.com (T.H.); fialova.kristyna@centrum.cz (K.F.); lukas.jelinek@vscht.cz (L.J.)

**Keywords:** hop, prenylflavonoids, desmethylxanthohumol, 8-prenylnaringenin, estrogenic activity, phytoestrogens

## Abstract

In recent years, the interest in the health-promoting effects of hop prenylflavonoids, especially its estrogenic effects, has grown. Unfortunately, one of the most potent phytoestrogens identified so far, 8-prenylnaringenin, is only a minor component of hops, so its isolation from hop materials for the production of estrogenically active food supplements has proved to be problematic. The aim of this study was to optimize the conditions (e.g., temperature, the length of the process and the amount of the catalyst) to produce 8-prenylnaringenin-rich material by the magnesium oxide-catalyzed thermal isomerization of desmethylxanthohumol. Under these optimized conditions, the yield of 8-prenylnaringenin was 29 mg per 100 gDW of product, corresponding to a >70% increase in its content relative to the starting material. This process may be applied in the production of functional foods or food supplements rich in 8-prenylnaringenin, which may then be utilized in therapeutic agents to help alleviate the symptoms of menopausal disorders.

## 1. Introduction

Female cones from hops, *Humulus lupulus* L., contain several important biologically active substances with proven beneficial effects on human health. The use of hops for medicinal purposes dates back many centuries [1,2]. Pharmaceutically important are polyphenolic compounds, especially prenylflavonoids, whose content can be up to 1.7 %wt. in the hop cones depending on the hop variety, harvest year and the agronomic conditions [3,4].

Prenylflavonoids are of particular interest due to their antioxidant [5,6], anti-carcinogenic [7,8,9,10], antimicrobial [11,12] and anti-inflammatory properties [13,14]. Moreover, prenylflavonoids are significant for their estrogenic potential [15,16,17]. Although the estrogenic effects of hops have been known in traditional medicine for a long time [18], the substance responsible for these effects has been identified only relatively recently [16]. The minor hop prenylflavonoid, 8-prenylnaringenin, is considered one of the most potent phytoestrogens currently known, exhibiting approximately 50 times greater estrogenic activity than other well-known phytoestrogens such as genistein or coumestrol [16]. The estrogenic activities of 8-prenylnaringenin are based primarily on its similar chemical structure to endogenous steroidal estrogens, thus resulting in its ability to interact with the estrogen receptors ERα and ERβ [16,19]. Additionally, 8-prenylnaringenin may also act as an enzyme effector. This flavanone stimulates alkaline phosphatase in Ishikawa Var I cells and the growth of the estrogen-dependent MCF-7 breast cancer cell line [20,21]. Moreover, 8-prenylnaringenin inhibits the activity of aromatase [22] that regulates the level of blood estradiol by converting androgens to estrogens [23]. Thus, due to its estrogenic abilities, it could partially attenuate the negative effects of breast cancer treatment with other synthetic aromatase inhibitors [22]. 

Due to the potential of plant substances with estrogenic effects to affect human health through their ability to act as alternatives to human steroidal hormones, attention has recently focused on their use as dietary supplements or in drugs used to alleviate the symptoms of menopausal discomfort [24] caused by insufficient production of estradiol during menopause [25]. These symptoms are usually treated by hormone replacement therapy (HRT), involving the application of relatively high doses of hormones [26]. On the one hand, this treatment eliminates menopausal discomforts (e.g., hot flashes, anxiety and insomnia) and reduces the risk of certain cardiovascular diseases and osteoporosis [27]. On the other hand, it can have negative effects, especially an increased risk of breast and uterine cancer and an increased risk of thrombosis due to blood clotting [17,27].

Studies dealing with 8-prenylnaringenin have primarily focused on its potential use in the prevention of climacteric symptoms, especially to inhibit osteoporosis [15]. It was demonstrated that 8-prenylnaringenin may increase bone mineral density in postmenopausal women and reduce the risk of fractures [28,29,30]. Due to the positive effects of prenylflavonoids on human health and the potential to use the estrogenically active prenylflavonoids as a complement to HRT, these natural substances could become an important supplement to conventional treatments of menopausal problems.

**Figure 1:**
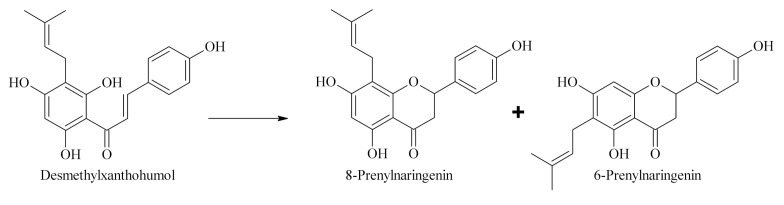
Isomerization of desmethylxanthohumol to 6-prenylnaringenin and 8-prenylnaringenin.

Food supplements and functional foods enriched in phytoestrogens for ensuring a balanced level of female hormones are already available on the market, but the content of estrogen-active substances is often unspecified and questionable [31,32]. Hops contain variable levels of prenylflavonoids, and the content of 8-prenylnaringenin in hop cones is generally very low (less than 0.01 %wt.) [33], so the estrogenic activities of other prenylflavonoids are almost negligible [34]. Therefore, there is demand for hop material enriched in 8-prenylnaringenin. One option may be through the isomerization of estrogenically inactive desmethylxanthohumol, whose content in hop cones is much higher (0.04–0.22 %wt.) [4] than 8-prenylnaringenin and can naturally produce a mixture of 6-prenylnaringenin and 8-prenylnaringenin (Figure 1) [35]. Unfortunately, the ratio of isomerization during the production of beer (which is the only source of hop prenylflavonoids in the human diet) is typically 3:2 in favor of 6-prenylnaringenin [36], whose estrogenic activity is less than 1% relative to 8-prenylnaringenin [34]. Therefore, a new approach is needed to form 8-prenylnaringenin. The crucial parameters in the preparation of hop material rich in estrogenically active prenylflavonoids are the maximization of desmethylxanthohumol content in the raw material and the determination of the appropriate conditions under which the isomerization equilibrium can be moved in favor of 8-prenylnaringenin.

In recent years, the hop-growing industry has focused on breeding new varieties with a higher content of biological active compounds. One of these varieties, Vital, bred and registered in the Czech Republic in 2008, has a high content of prenylflavonoids, especially desmethylxanthohumol (0.3–0.4 %wt. in the hop cones), which is approximately twice that of other varieties [4]. Therefore, his hop variety is suitable as a starting material for pharmaceutical applications and as a starting material for this study [37].

The aim of this study was to determine the appropriate conditions for the isomerization of hop desmethylxanthohumol to achieve a maximum yield of 8-prenylnaringenin. On an industrial scale, this material may then be used in the production of dietary supplements that can be used to alleviate the symptoms of menopausal discomfort. Our isomerization procedure was carried out in powder (spent hops after CO_2_ extraction) rather than a solution and uses food-grade MgO, which allows us to apply lower temperatures. Moreover, there is no isomerization of xanthohumol to isoxanthohumol at these lower temperatures.

## 2. Results and Discussion

To the best of our knowledge, there are only a few techniques for transforming some of the hop prenylflavonoids into estrogen-active 8-prenylnaringenin. Possemiers and colleagues [38] invented and patented the demethylation of isoxanthohumol by *Eubacterium limosum* in Brain Heart Infusion (BHI) broth with a conversion of up to 50% and up to 90% by using specially selected strains [39]. Fu et al. [40] used fungi for the demethylation of isoxanthohumol with a conversion of up to 1.5%. Thus, there is a big difference between the yield of conversion of bacteria and fungi. There is also a second approach by organic synthesis where pure substances are used for conversion, which is based on either the efficient synthesis of the phytoestrogen 8-prenylnaringenin from isoxanthohumol with magnesium iodide etherate [41] or the synthesis of the mixture of 8-prenylnaringenin and 6-prenylnaringenin from xanthohumol using lithium chloride (LiCl), dimethylformamide (DMF) and microwave irradiation (MW) [42]. There is also an alternative in the application of metal oxides as a catalyst in the conversion of xanthohumol to 8-prenylnaringenin [43]. However, the industrial application of this biotransformation process is associated with a number of issues. The microorganism used belongs to risk class 2 according to the German Federal Institute of Occupational Safety and Health [44], and the trials were also carried out only with pure substances. Therefore, the industrial application of this procedure for the production of medicinal preparations or food supplements with estrogenic activity is likely to be associated with additional costs that are associated with preparing pure isoxanthohumol or xanthohumol and purification of the product. Our procedure uses edible MgO as a catalyst for the conversion of desmethylxanthohumol to 8-prenylnaringenin directly in the spent hops after CO_2_ extraction. This conversion is highly suitable for hop varieties with a very high content of desmethylxanthohumol, such as the hop variety Vital of Czech origin. The resulting materials after conversion contain a high content of 8-prenylnaringenin, which can be used directly in the production of food supplements for the relief of menopausal symptoms.

From this point of view, a simple microbial-free physicochemical process that exploits hops, or even better, the residual material from the production of hop pellets or CO_2_ extracts, has great potential.

### 2.1. Analysis of Starting Hop Material

The starting material used in this study was pellets produced as a residue after the supercritical CO_2_ extraction of hops (variety Vital). This material does not yet have any industrial use. The contents of the main hop prenylflavonoids (xanthohumol, isoxanthohumol, 6-prenylnaringenin, 8-prenylnaringenin and desmethylxanthohumol) determined by HPLC-PDA and expressed as mg/100 g of dry matter (DW) are summarized in Table 1. The predominant prenylflavonoid in the material was xanthohumol (920.2 mg/100 gDW), and the content of estrogenically active 8-prenylnaringenin and its precursor desmethylxanthohumol were 17.1 mg/100 g and 97.4 mg/100 gDW, respectively. The 8-prenylnaringenin content was higher than in a previous study [45] in the hop variety Magnum (4.51 mg/100 gDW), as well as in hop residues after CO_2_ extraction (10.8 mg/100 gDW).

In addition, extraction with nonpolar supercritical CO_2_ led to the removal of most hop resins [46], particularly α-bitter acids that could cause undesirable bitter tastes [47]. The final content of these substances in the hop residue was about 1 %wt., whereas the initial content in the Vital hop cones ranged from 10 to 15 %wt. [48]. As a residue from the production of raw material used in the brewing industry, this material does not represent a risk with regard to toxicity even at high doses, as was confirmed for all major groups of hop components [1].

These data show that the residue after CO_2_ extraction of the hop variety Vital is an appropriate and economically beneficial raw material for the production of food supplements with increased estrogenic activities.

### 2.2. Determination of Appropriate Conditions for the Isomerization of Prenylflavonoids

The rate of the isomerization of prenylflavonoids and structurally related hop resins is dependent on many physicochemical parameters, in particular temperature, pH, the amount of the catalyst and the length of time for the isomerization process [49,50,51]. The isomerization procedure was performed without the presence of oxygen (i.e., wrapped and vacuumed).

#### 2.2.1. Influence of Temperature

**Figure 2:**
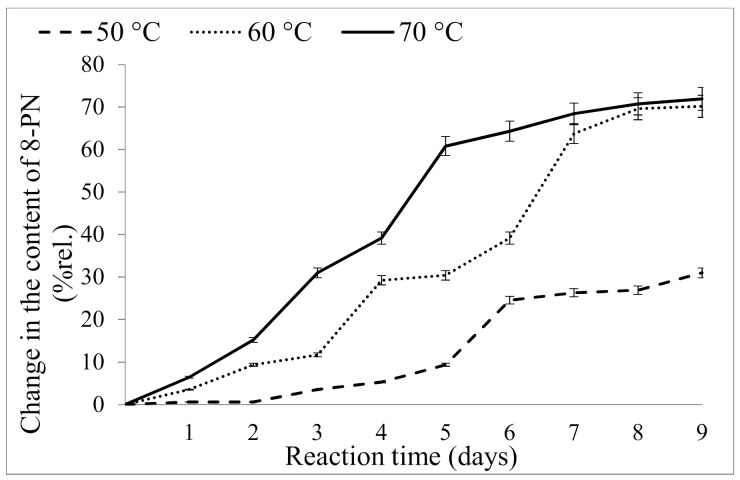
Isomerization of desmethylxanthohumol to 8-prenylnaringenin in the spent hops at different temperatures.

The catalyst was mixed with the hop material (0.3 %wt.), and a series of samples were heated in the oven for 9 days at different temperatures (50 °C, 60 °C and 70 °C). These temperatures were chosen to avoid the isomerization of xanthohumol to isoxanthohumol and the decomposition of 8-prenylnaringenin at temperatures greater than 80 °C [52]. The average relative changes in the content of 8-prenylnaringenin in the material are shown in Figure 2. The initial concentration of 8-prenylnaringenin was at time 0, and during the total time, the content of 8-prenylnaringenin was increased (expressed in relative %).

For the samples exposed to a low temperature (50 °C), an approximate 31 %rel. increase in the content of 8-prenylnaringenin was observed (corresponding to a change from 17.1 to 22.4 mg/100 gDW). The greatest increase in 8-prenylnaringenin was observed after exposure to a temperature of 70 °C. Under these conditions, the final content of 8-prenylnaringenin (29.1 mg/100 gDW) represented a 72% increase relative to the content in the starting material. The yield of 8-prenylnaringenin in the hop material isomerized at 60 °C was slightly lower (Figure 2). Extensive degradation of 8-prenylnaringenin occurred at temperatures above 70 °C. After 4 days of isomerization at 80 °C, the level of 8-prenylnaringenin was below the limit of detection for the method.

Therefore, the isomerization of other prenylflavonoids to 8-prenylnaringenin was strongly dependent on temperature, which was similar to the reactions of other nonpolar hop components [36,49]. At low temperatures, the transformation was also low, but very high temperatures led to an undesirable decomposition of the products resulting in reduced yields of 8-prenylnaringenin and other prenylflavonoids [52]. For an industrial-scale technological process, it is important to consider economic factors. Energy demands for the isomerization of the material at 70 °C would be significantly higher than at 60 °C, and it would therefore be necessary for a producer to make decisions based on an overall economic evaluation of the process.

#### 2.2.2. Influence of Reaction Time

**Figure 3:**
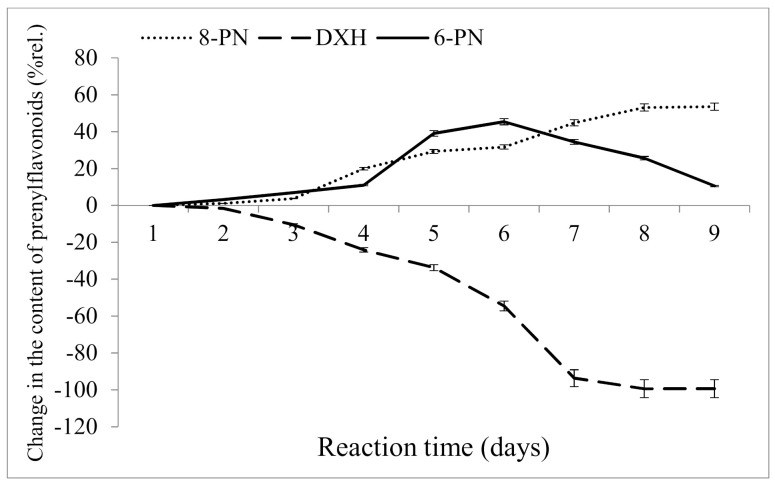
Isomerization of desmethylxanthohumol to 6-prenylnaringenin and 8-prenylnaringenin in the spent hops after CO_2_ extraction.

The samples were mixed with the catalyst (0.3 %wt.) and left in the oven for 9 days at 60 °C. The results (Figure 3) show that beginning from day 6, there was a shift in the ratio between 6-prenylnaringenin and 8-prenylnaringenin in favor of the latter. The content of 8-prenylnaringenin increased slightly even after the complete conversion of desmethylxanthohumol, suggesting that the conversion of 6-prenylnaringenin to 8-prenylnaringenin had occurred.

**Figure 4:**
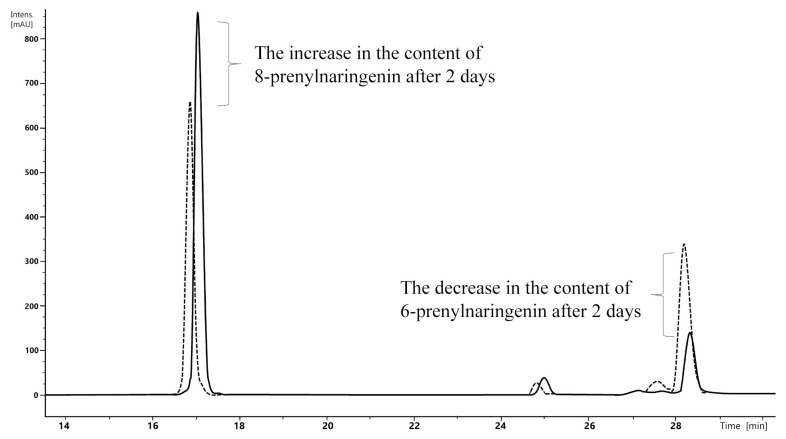
Chromatogram showing changes in levels of 8-prenylnaringenin and 6-prenylnaringenin, measured at λ = 290 nm in standard solutions over a 2-day period.

To verify this, a mixture of prenylnaringenin standards was exposed to model conditions (catalysis at a concentration of 0.3 %wt., temperature of 60 °C, incubation period 2 days), and the concentrations of the two isomers in solution were determined. The analysis showed that 6-prenylnaringenin was converted to 8-prenylnaringenin (Figure 4).

Based on data obtained by analyzing the changes in 8-prenylnaringenin, as illustrated in Figure 3, an 8-day reaction time was chosen as suitable for the isomerization of desmethylxanthohumol at a temperature of 60 °C. A longer isomerization time (9 days) would not be economically viable for manufacturers due to the higher costs, which are compensated only by a negligible increase in the content of 8-prenylnaringenin.

#### 2.2.3. Influence of Magnesium Ions

According to the available literature, the isomerization of some hop compounds is pH-dependent [53], where a higher pH stimulates the reaction rate. A similar reaction is commonly used for the production of iso-pellets, a brewing bittering material in which α-bitter acids are converted into iso-α-bitter acids by the addition of a small quantity of food grade magnesium oxide (1–3 %wt.) or hydroxide at a temperature of 50 °C, which increases the pH and acts as a catalyst [54]. Magnesium oxide was chosen in our study to accelerate the isomerization of desmethylxanthohumol to 8-prenylnaringenin. The aim was to identify the appropriate amount of magnesium oxide that needed to be added to the hop material.

**Figure 5:**
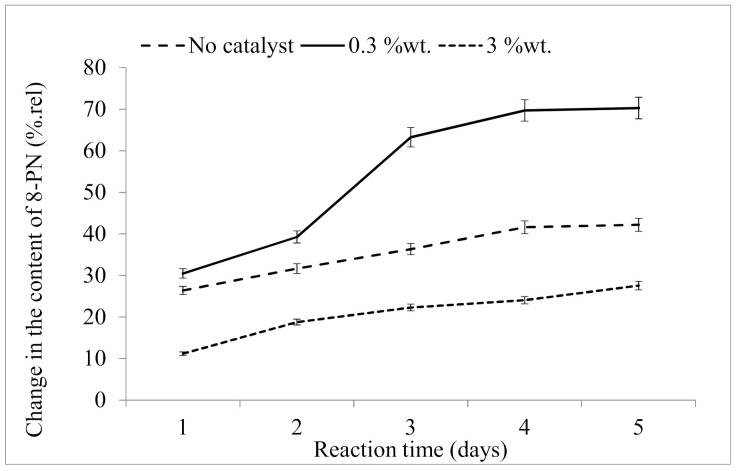
Catalyst-dependent changes in the content of 8-prenylnaringenin (8-PN) relative to the content in starting material.

The data suggest (Figure 5) that the most appropriate amount of the catalyst was 0.3 %wt. of the initial material. By day 8, the increase in 8-prenylnaringenin content was almost 70 %rel.

The use of greater amounts of the catalyst was unsatisfactory in terms of the yield of 8-prenylnaringenin, where heating the mixture with 3 %wt. of magnesium oxide led only to a 24 %rel. increase in 8-prenylnaringenin over the 8-day period. Even the isomerization performed without a catalyst was significantly better in this respect (41.6% increase).

**Figure 6:**
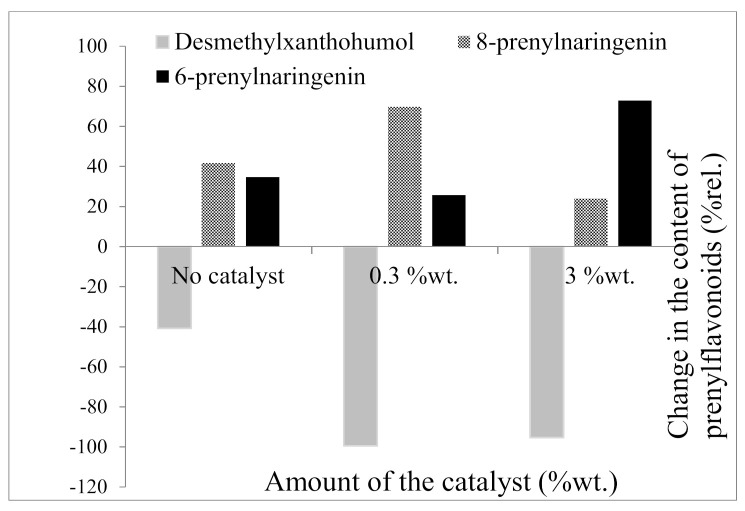
Catalyst-dependent changes in the content of prenylflavonoids after 9 days of isomerization relative to the content in starting material.

As is clear from Figure 6, in the case of the 3 %wt. catalyst the lower content of 8-prenylnaringen was due to a shift in the balance of products toward 6-prenylnaringenin. We assumed that the higher level of the catalyst significantly accelerated the conversion of desmethylxanthohumol to 6-prenylnaringenin but also inhibited the conversion of 6-prenylnaringenin to 8-prenylnaringenin. The addition of the 3 %wt. catalyst caused a 72.8 %rel. increase in the content of 6-prenylnaringenin in the final product (on day 9 of isomerization) compared to samples without the addition of the catalyst (34.7 %rel.).

## 3. Materials and Methods

### 3.1. Chemicals

All the chemicals used were of HPLC gradient or analytical grade. Diethyl ether was purchased from Lach-Ner, Czech Republic. Acetonitrile was purchased from Sigma-Aldrich, Germany. Hydrochloric acid (38%) and formic acid were purchased from Penta, Czech Republic. The xanthohumol standard (95%) was purchased from Hopsteiner, Germany. Standards of isoxanthohumol, 6-prenylnaringenin and 8-prenylnaringenin were purchased from Toroma Organics Ltd., Germany. Milli-Q water was used for HPLC analysis. Magnesium oxide light (BP, Ph. Eur., USP, E 530) extra pure, pharma grade was purchased from Merck Life Science Ltd., Prague, Czech Republic.

### 3.2. Hop Material

Residual hop material was prepared by Flaveko Trade, Czech Republic, from the hop variety Vital using supercritical carbon dioxide extraction (50 °C, 290 bar). The consistency of the hop residue after extraction was powdery, and this material was used for the isomerization experiments.

### 3.3. Isomerisation of Prenylflavonoids in Hop Material

From the original material, which was first homogenized on a laboratory homogenizer, about 10 g was weighed and then the appropriate amount (0.3–3 %wt.) of magnesium oxide catalyst was added. This mixture was thoroughly homogenized and subsequently transferred to double-sided plastic foil from which air was sucked out and sealed by means of a vacuum foil welder. The prepared material was then placed in an oven for 1–10 days at adjusted temperatures (50, 60 and 70° C). After the completion of the isomerization reaction, the material was analyzed according to the procedure described in part 3.4.

### 3.4. Preparation of Samples and HPLC-PDA Analysis

Analyses of prenylflavonoids (xanthohumol, isoxanthohumol, 6-prenylnaringenin and 8-prenylnaringenin) in hop material were performed using a method commonly used for the determination of α- and β-bitter acids in hops based on the extraction with a diethyl ether–methanol mixture and the subsequent analysis by an HPLC with photodiode array detection [55]. This method was slightly modified to achieve better yields and the separation of the desired prenylflavonoids.

The hop material was homogenized in a laboratory mill, 1 g was weighed accurately to four decimal places and was then extracted with 20 mL of methanol and 100 mL of diethyl ether for 30 min on a shaker. Subsequently, 40 mL of 0.1 M hydrochloric acid were added, and the extraction was continued for another 10 min. Then, 20 mL of the diethyl ether phase was transferred to a 100 mL flask and evaporated to dryness. After evaporation, the extract was diluted with 5 mL of methanol, filtered through a 0.45 µm PTFE filter and transferred to a vial.

HPLC analyses were performed at 25 ± 1 °C. The samples (10 µL injected volume) were analyzed using an Agilent 1100 series system (Agilent Technologies, Santa Clara, CA, USA) equipped with a photodiode array detector (PDA). Separation was achieved using an ECLIPSE XDB-C18 column (5 μm, 4.6 × 150 mm; Agilent, Santa Clara, CA, USA). The mobile phase consisted of water with 0.05% formic acid (solvent A) and acetonitrile with 0.05% formic acid (solvent B). The flow rate was 0.8 mL/min, and the solvent composition varied as follows: 0–40 min, 35–62% B; 40–42 min, 62–95% B; 42–47 min, 95% B. The HPLC-PDA analysis was performed by monitoring two different wavelengths: 290 nm for isoxanthohumol, 6-prenylnaringenin and 8-prenylnaringenin and 370 nm for xanthohumol.

### 3.5. HPLC-PDA Method of Analysis Validation

The HPLC method validation parameters (repeatability, limit of detection (LOD) and limit of quantification (LOQ)) were determined by repeating the process of isolation and analysis of prenylflavonoids (xanthohumol, isoxanthohumol, desmethylxanthohumol, 8-prenylnaringenin and 6-prenylnaringenin) eight times. The repeatability of the method was expressed as the relative standard deviation (RSD). The RSD for xanthohumol, isoxanthohumol and 8-prenylnaringenin was 4.9%, 4.5% and 3.7%, respectively. The LOD of this method for 8-prenylnaringenin was 0.713 mg/100 g, and the LOQ was 2.375 mg/100 g. This method can be considered reliable and is therefore suitable for the determination of prenylflavonoids in hop material.

### 3.6. Statistics

All results were expressed as the average values of three experiments that were repeated independently.

## 4. Conclusions

The aim of this study was to define suitable conditions for the optimal conversion of estrogenically inactive desmethylxanthohumol to the effective phytoestrogen, 8-prenylnaringenin. The starting material for the experiments was the residual material of the hop variety Vital after extraction with supercritical carbon dioxide, and the isomerization reaction took place in solid state.

The effects of temperature, the length of the process and the amount of the catalyst were studied, and the appropriate conditions were identified as:

- Isomerization for 8 days;
- A temperature of 60 °C;
- The use of a 0.3 %wt. magnesium oxide catalyst.

By applying these parameters, a product containing 29 mg of 8-prenylnaringenin per 100 gDW of hop material was prepared. This represented a more than 70% increase relative to the content of 8-prenylnaringenin in the starting material. Unlike the previously published procedures, this simple method does not involve the risks associated with the use of a potentially pathogenic micro-organism. The production costs should also be significantly lower, as costs associated with the preparation of the culture medium and isolation of the finished product are eliminated. The prepared material can be used directly as a component of functional foods or dietary supplements, developed specifically to help alleviate menopausal problems [56]. Where this material may be used for the production of dietary supplements, a 0.8 g capsule would contain 232 µg of 8-prenylnaringenin, which is comparable to commercially available products used to alleviate menopausal discomfort. In addition, other prenylflavonoids with proven health-promoting properties (i.e., xanthohumol and isoxanthohumol) are preserved in the material, thus representing an additional benefit that is associated with the use of products made from this material.

This procedure was used in the production of the food supplement MenoPrima Bella^®^ for the company Biomedica (https://www.bio-medica.cz/, accessed on 6 October 2021).

## Figures and Tables

**Table 1:** Characterization of the starting hop material.

Prenylflavonoid	(mg/100 gDW)
Desmethylxanthohumol	97.4
8-Prenylnaringenin	17.1
6-Prenylnaringenin	46.7
Xanthohumol	920.2
Isoxanthohumol	29.4
α-Bitter acids	1004
β-Bitter acids	n.d

Moisture (%wt.) 6.2.
